# Expression Profile of *IL-2*, *IL-6*, *IL-10*, and *TNF-α* in Breast Tumors

**DOI:** 10.3390/ijms26167841

**Published:** 2025-08-14

**Authors:** Harryson W. G. dos Santos, Beatriz C. Bramante, Matheus M. Perez, Glaucia L. da Veiga, Beatriz da C. A. Alves, Fernando L. A. Fonseca

**Affiliations:** 1 Laboratório de Análises Clínicas, Centro Universitário FMABC, Santo André 09060-650, Brazil; harrysongodoy@yahoo.com.br (H.W.G.d.S.); biabramante@hotmail.com (B.C.B.); matheusmpontoperez@gmail.com (M.M.P.); grlveiga@gmail.com (G.L.d.V.); bcaalves@uol.com.br (B.d.C.A.A.); 2 Departamento de Ciências Farmacêuticas, Universidade Federal de São Paulo, Diadema 09913-030, Brazil

**Keywords:** neoplasm, breast, inflammation, cytokines, gene expression

## Abstract

Chronic inflammation is associated with several neoplasms. Many studies tried to evaluate the correlation between cytokines and the pathogenesis of various cancer types and *IL-2*, *IL-6*, *IL-10*, and *TNF-α* are often target of these analyses. The aim of the present study was to analyze cytokines mRNA expression in breast cancer samples to better understand pathogenesis and clinical aspects. Patients were selected from the oncology service of Centro Universitário FMABC; tumor RNA was obtained from formalin-fixed paraffin-embedded biopsies of breast cancer tissue. Gene expression was assessed by qPCR. Samples from 95 patients were obtained, presenting tumor stages varying from 0 to IIIB, with most of them in stage IIIA (33.68%). *IL-2* and *TNF-α* expression presented a significant correlation with tumor stage. There was no correlation of cytokines expression with Ki-67 and prognostic factors. The study illustrated the pleiotropic role of *IL-2*, with no expression in early stages of cancer, varying according to “stage worsening”. Regarding progesterone receptor (PR), correlation with *TNF-α* and *IL-2* can reinforce the role of PR as an indicator of positive prognosis. The findings of this investigation suggest *IL-2* and *TNF-α* could be evaluated in a larger study to better understanding pathogenesis and prognosis for this patient profile.

## 1. Introduction

Cancer is a leading cause of death worldwide, accounting for nearly 10 million deaths in 2020 [1]. Breast cancer is the most frequently diagnosed cancer and is the principal cause of cancer death among females globally, with a mortality rate of more than 500,000 annually. The 2018 Global Cancer Survey indicates that breast cancer has the highest incidence of malignant tumors among women worldwide, accounting for 15% of all malignant tumor deaths [2,3].

Over a hundred years ago, a connection between cancer development and sites of chronic inflammation was first suggested [4]. Today, this association is well-established, with numerous studies demonstrating that chronic inflammation contributes to the onset and progression of various human cancers. Cancerous tissues can produce pro-inflammatory cytokines and immunomodulatory factors that support tumorigenic processes such as proliferation, tissue invasion, and metastasis [5]. In breast cancer specifically, cytokines have been widely recognized as essential mediators of intercellular communication, influencing cell proliferation, survival, differentiation, immune responses, and tumor advancement [6,7]. Notably, reduced inflammatory activity has been linked to better outcomes in early-stage breast cancer patients [8,9], possibly by creating a tumor microenvironment that is less conducive to progression and dissemination [10].

Breast cancer can be classified using both histological and immunopathological criteria. The two predominant histological forms are invasive ductal carcinoma (IDC) and invasive lobular carcinoma (ILC), which together account for approximately 90% of all diagnoses. Immunopathological classification is based on the expression of estrogen receptor (ER), progesterone receptor (PR), and the human epidermal growth factor receptor 2 (HER2), which are key markers used to inform prognosis and therapeutic strategies. Additionally, intrinsic molecular subtypes identified through mRNA profiling include luminal A, luminal B, HER2-enriched, basal-like, and normal-like subtypes [11,12].

Cytokines have diverse and significant functions in breast cancer biology, contributing to processes ranging from tumor initiation to metastatic spread [13]. Among them, interleukin-6 (*IL-6*) is a pro-inflammatory cytokine with well-known roles in immune regulation, inflammatory responses, and hematopoiesis [14]. *IL-6* also influences multiple tumor-related mechanisms, such as apoptosis inhibition, enhancement of cell proliferation, migration, invasion, neovascularization, and metastasis [15]. Interleukin-10 (*IL-10*), often described as a cytokine with inhibitory effects, plays a pivotal role in modulating inflammation and infectious disease outcomes [16], and it is also involved in the suppression of pro-tumor inflammatory responses [17], as well as the regulation of tumor-associated angiogenesis [18]. Another cytokine, interleukin-2 (*IL-2*), contributes to the activation of the immune system’s anti-tumor response, facilitating the elimination of malignant cells [19]. Tumor Necrosis Factor Alpha (*TNF-α*), despite its name, can enhance tumor growth and dissemination by promoting migration within the tumor microenvironment [20]. Nonetheless, the precise relationship between molecular subtypes of breast cancer and their distinct cytokine secretion profiles has yet to be fully clarified [21].

## 2. Results

Tumor samples from 95 women were included in this study. Patients’ characteristics are shown in Table 1. They had different tumor types, and the stages varied from 0 to IIIB, with the majority in stage IIIA (33.68%). The ages of the subjects ranged from 31 to 89 years, with a mean of approximately 56 years.

The correlation of inflammatory cytokines and tumor characteristics can be found in Table 2. No correlation was detected between *IL-6* and any variable. The cytokines *IL-2* and *TNF-α* showed a significant correlation with tumor stage. The expression of *IL-2* was not detected in the early stages of the tumor, but it progressively increased as the stage advanced. *TNF-α* was almost absent in samples from stages 0 to IIIB. Eighty percent of the samples showed positivity for PR, which is consistent with the distribution of sample stages and good prognosis. For this receptor, *TNF-α* was the cytokine with the highest correlation, whereas *IL-2* showed a trend of correlation. The expression of *IL-10* could not be detected in the samples included in this study. Cytokine expression showed no correlation with age or the prognostic factors evaluated by immunohistochemistry (Ki-67) (Table 3).

## 3. Discussion

Several studies employing varied methodologies and analyzing heterogeneous sample types, such as tumor tissues and peripheral blood, have been conducted to explore the involvement of different cytokines in tumor development and progression [21,22]. In the context of inflammation, it is well-established that inflammatory cytokines present within the tumor microenvironment can influence multiple stages of cancer progression, including initiation, proliferation, and promotion, among others [4]. Given the high incidence of metastasis and the often unfavorable prognosis despite standard interventions such as surgery, chemotherapy, and radiotherapy, identifying new biomarkers may improve prognostic assessments [23,24]. In this study, we investigated the expression levels of *IL-2*, *IL-10*, *IL-6*, and *TNF-α* and assessed their associations with clinical and pathological tumor features. These findings may provide insight into their potential prognostic value and relevance for future therapeutic strategies.

Although numerous studies have indicated possible associations between *IL-6* and tumor-related factors, including estrogen receptor (ER) status [25], hormonal regulation of *IL-6* production—particularly by estrogen [26]—and links between elevated serum *IL-6* levels and unfavorable prognosis, our findings did not reveal any significant correlation between *IL-6* and the clinical variables analyzed. These results are in agreement with previous research involving large breast cancer cohorts, which similarly assessed serum *IL-6* levels and found no strong associations [5].

*IL-2* is primarily produced by antigen-stimulated CD4+ T cells, although its expression can also be induced in CD8+ T cells, natural killer (NK) cells, and activated dendritic cells (DCs) [27,28]. Due to its role in promoting T cell proliferation, *IL-2* is often referred to as “T-cell growth factor” (TCGF) [29], which supports its application in cancer immunotherapy. In our study, we identified a notable trend suggesting a relationship between *IL-2* expression and tumor stage. Notably, *IL-2* was absent in early-stage tumors. As tumor stage advanced, *IL-2* expression appeared to fluctuate in a non-linear fashion, demonstrating a pattern consistent with increasing tumor severity. This complex behavior may reflect the multifaceted nature of *IL-2* in immune regulation, as it influences multiple immune cell types across different phases of tumor progression [26], potentially through mechanisms involving the activation of the VEGF signaling pathway.

A pro-inflammatory immune profile, particularly when accompanied by infiltration of cytotoxic lymphocytes into the tumor microenvironment, has been associated with improved prognosis across multiple cancer types, including breast cancer [19]. *IL-2*, in cooperation with cytokines such as IL-15, plays a pivotal role in enhancing the anti-tumor functions of natural killer (NK) cells. It contributes to immune regulation by improving tumor antigen presentation and augmenting the activity of macrophages and NK cells [29]. Furthermore, elevated *IL-2* levels in cases of breast cancer with bone metastases have been linked to the activation of anti-tumor immune responses [30]. Within this framework, the *IL-2* expression patterns observed in our study suggest that patients exhibiting this cytokine profile may have a more favorable prognosis. The increased *IL-2* expression in later tumor stages could reflect a compensatory immune mechanism aimed at limiting metastatic progression [30].

*TNF-α* is a central mediator in the inflammatory cascade, initiating the production of other pro-inflammatory cytokines that recruit and activate immune cells to sites of tissue injury, thereby facilitating repair processes. However, when its expression becomes chronic, *TNF-α* may shift roles and support tumor development by contributing to tissue remodeling, angiogenesis, and facilitating tumor growth and metastasis. Recent findings highlight its involvement in carcinogenesis through the upregulation of genes related to proliferation, apoptosis, inflammation, metastasis, and angiogenic signaling [5]. In our investigation, *TNF-α* expression was more frequently associated with non-malignant tumor features. Specifically, we observed a significant correlation between lower tumor stages (stage 0 to IIIB) and the absence of *TNF-α* gene expression, suggesting its limited involvement during earlier stages of disease progression.

It is well known that PR is currently used as a positive prognostic factor and to predict therapy response [31]. Furthermore, there is a consensus that different cytokine expressions are inversely correlated with ER and PR status in breast cancer [31]. Our study revealed a significant correlation between PR and *TNF-α*, as 100% of positive PR samples did not express *TNF-α*, whereas about 10% of samples with *TNF-α* positivity were PR-negative. For *IL-2*, although the relationship was not significant, about 65% of PR-positive samples were *IL-2*-negative. These findings support the rationale that PR plays a role in anti-inflammatory and immunosuppressive reactions, as seen in the relationship between *NF-κB* and PR [31].

Finally, our work showed no significant relationship between patient age, Ki-67, and *IL-2*, *IL-6*, and *TNF-α*. Although it is well known that breast cancer incidence increases with age [32], we failed to demonstrate a relationship between these cytokines as prognostic markers and age. However, since Ki-67 is associated with poor breast cancer prognosis [32] and our study showed that *IL-2* levels correlated with tumor aggressiveness, the relationship between Ki-67 and *IL-2* in breast cancer tumors warrants further investigation.

The results presented in this work could contribute to the understanding of the inflammatory profile of breast cancer and its prognostic predictions, as recent studies have identified immunological parameters and their relation to disease outcomes [19]. Additionally, since tumor infiltration by cytotoxic lymphocytes is often orchestrated by chemokines expressed within the tumor microenvironment [19], the identification and quantification of cytokines could enhance cancer prognostic tools.

Triple-negative breast cancer (TNBC) is recognized as the most aggressive form of breast cancer and is defined by the lack of expression of estrogen receptor (ER), progesterone receptor (PR), and human epidermal growth factor receptor 2 (HER2) [32]. This subtype is further divided into six molecular classifications: basal-like 1 (BL1), basal-like 2 (BL2), mesenchymal (M), mesenchymal stem-like (MSL), immunomodulatory (IM), and luminal androgen receptor (LAR). These subtypes are characterized by either dysregulated gene expression profiles or the activation of specific signaling pathways and receptors. Notably, the M and IM subtypes show marked upregulation of the *IL-12* and *IL-7* signaling pathways. However, the present study did not include analysis of these particular cytokines [32].

Menopausal status was not collected, which could influence ER and PR. The study focuses on a small group of cytokines, and further research could investigate other molecules linked to breast cancer, such as *IL-17*. Since the study uses only PCR techniques, additional analyses, such as immunohistochemistry, could be employed to confirm the results. The expression of *GAPDH* can be affected by factors such as oxygen availability, cellular developmental stage, and type of tissue; although the results of the present research are coherent, other housekeeping genes could have been used.

One limitation of this study is the restricted panel of cytokines analyzed. We focused on *IL-2*, *IL-6*, *IL-10*, and *TNF-α* due to their well-documented relevance in breast cancer immunobiology, as reported in previous studies [20]. These cytokines are considered key mediators of the immune and inflammatory responses in this disease context. However, we acknowledge that including additional cytokines could have provided a more comprehensive view of the inflammatory landscape. Given the exploratory nature of this phase and the limited quantity of biological material (mRNA) extracted from tumor sections, we prioritized a targeted approach aligned with our central hypothesis. Furthermore, the lack of detectable expression of certain cytokines still offers valuable insight, possibly indicating a lack of involvement or downregulation in this specific model. Future studies with larger sample volumes and broader cytokine panels are warranted to deepen the understanding of the tumor immune microenvironment.

## 4. Materials and Methods

### 4.1. Eligibility Criteria

#### 4.1.1. Patient Selection

Ninety-five patients were selected from the oncology service at Centro Universitário FMABC, all of whom had confirmed breast cancer through anatomopathological examination and had undergone mastectomy. The selected patients ranged in age from 30 to 90 years.

#### 4.1.2. Inclusion Criteria

Inclusion criteria required that patients be women without non-neoplastic mammary comorbidities, without other neoplasms, and without chronic and/or inflammatory diseases such as HIV (Human Immunodeficiency Virus), hepatitis, and diabetes.

#### 4.1.3. Exclusion Criteria

Exclusion criteria applied to women with non-neoplastic breast comorbidities, other neoplasms, or chronic and/or inflammatory diseases such as HIV (Human Immunodeficiency Virus), hepatitis, and diabetes. Any women who did not meet all the inclusion criteria or who refused to participate were also excluded from the study.

#### 4.1.4. Samples

Tumor biopsy samples were collected from each patient, and the tissue was prepared as paraffin-embedded. Clinical and histopathological data, including age, tumor stage, PR and ER status, and the proliferation index Ki-67, were recorded. All techniques followed good clinical laboratory analysis practices and the principles of the Helsinki Declaration. The patients signed an Informed Consent Form before the biopsies, and the study was approved by the Centro Universitário FMABC Ethics Committee (346712/14 of August 2018). This study was conducted in 2019 and 2020.

### 4.2. Gene Expression

The isolation of total RNA was performed from two or three sections of 10 µm obtained from each paraffin-embedded tumor sample using the RNeasy^®^ FFPE Kit (Qiagen, Valencia, CA, USA), with xylene as the deparaffinization agent, applying validated protocols. The kit protocol steps were strictly followed to ensure the integrity of the material. cDNAs were prepared from 1 µg of total RNA using the QuantiTect Reverse Transcription Kit (Qiagen, cat. no. 205311) according to the manufacturer’s protocol.

The expression of *IL-2*, *IL-10*, *IL-6*, and *TNF-α* genes was evaluated using Real-Time Polymerase Chain Reaction (qPCR). The specific primers were designed using Primer3 Input 0.4.0, available at https://bioinfo.ut.ee/primer3-0.4.0/ (accessed on 20 April 2024). The specificity of each primer was verified using Primer-BLAST, available at http://www.ncbi.nlm.nih.gov/tools/primer-blast (accessed on 20 April 2024) (Table 4). Amplification reactions were performed in a final volume of 15 µL containing 1X SYBR Green mix (QuantiTect SYBR Green PCR Kit, QIAGEN cat. no. 204054), 10 pmol of each specific primer, and 2 µL of cDNA. The cycling parameters included an initial hot start step at 95 °C for 10 min, followed by 40 repetitions of 95 °C for 15 s and 60 °C for 25 s.

### 4.3. Sample Size

The sample size was selected based on a pilot test, using GPower^®^ software (v. 3.1). With an alpha risk of 5%, a beta risk of 5%, and an actual power of 0.9512, the determined sample size was at least 45 individuals.

### 4.4. Statistical Analysis

To describe qualitative variables, absolute and relative data were used. For quantitative variables, data were presented as mean ± standard deviation (SD) for those with normal distribution (Shapiro–Wilk > 0.05) and as median (95% CI) for those without normal distribution (Shapiro–Wilk < 0.05). The association test for diagnostic variables was measured using the Pearson Chi-squared test. For the association of age and Ki-67 with inflammatory variables, the Spearman test was used. Ki-67 is a protein frequently used as an indicator of tumor proliferation. For all analyses, the confidence interval was set at 95%. The software used was Stata^®^ version 11.0.

## 5. Conclusions

Our results suggest that *IL-2* and *TNF-α* should be evaluated in a larger study to better understand their role in pathogenesis and prognosis, as they showed a significant relationship or a trend of relationship with tumor stage and PR. Once standardized and extensively tested, studies like this have the potential to be used in clinical practice, expanding diagnostic rates and anticipating treatment actions, thereby contributing to improved positive outcomes in breast cancer.

## Figures and Tables

**Table 1:** Sample characteristics.

Variable	*n*	%
Tumor type		
Invasive Ductal Carcinoma	77	81.05
Mucinous Carcinoma	3	3.16
In Situ Ductal Carcinoma	4	4.21
Invasive Ductal Carcinoma/PAGET	2	2.11
Invasive Lobular Carcinoma	7	7.37
Lobular Carcinoma	2	2.1
Stage		
0	2	2.11
IA	16	16.84
IIA	31	32.63
IIIA	32	33.68
IIB	4	4.21
IIIB	10	10.53
Her2-neo		
Negative	59	62.11
+	20	21.05
++	6	6.32
+++	10	10.53
PR		
Negative	19	20.00
Positive	76	80.00
ER		
Negative	16	16.85
Positive	79	83.15
Chemotherapy		
No	4	4.21
Yes	91	95.79
*IL2* mRNA		
Expression detected	65	68.42
Expression not detected	30	31.58
*IL6* mRNA		
Expression detected	87	91.58
Expression not detected	8	8.42
*IL10* mRNA		
Absent	95	100
*TNF-a* mRNA		
Expression detected	93	97.89
Expression not detected	2	2.11
	Mean (SD)	Range
Age	56.87 (13.63)	31.00–89.00
	Median	CI 95%
Ki-67 (%)	2.0	0.0; 10.0

SD: standard deviation. CI 95%: confidence interval 95%. PR: progesterone receptor; ER: estrogen receptor. +/3+, ++/3+, and +++/3+ represent standard positivity scores.

**Table 2:** Association of diagnosis and inflammatory variables.

Variables	*IL-2*		*p* *	*IL-6*		*p* *	*TNF-α*		*p* *
	No Expression	Expression		No Expression	Expression		No Expression	Expression	
	*n* (%)			*n* (%)			*n* (%)		
ER									
Negative	13 (81.25)	3 (18.75)	0.215	15 (93.75)	1 (6.25)	0.722	15 (93.75)	1 (6.25)	0.210
Positive	52 (65.83)	27 (34.17)		72 (91.13)	7 (8.87)		78 (98.73)	1 (1.27)	
PR									
Negative	16 (84.21)	3 (15.79)	0.098	18 (94.74)	1 (5.26)	0.579	17 (89.47)	2 (10.53)	0.004
Positive	49 (64.47)	27 (35.53)		69 (90.79)	7 (9.21)		76 (100)	0 (0.0)	
Chemo									
No	2 (50.00)	2 (50.00)	0.418	3 (75.00)	1 (25.00)	0.222	4 (100)	0 (0.0)	0.764
Yes	63 (69.23)	28 (30.77)		84 (92.31)	7 (7.69)		89 (97.80)	2 (2.20)	
Stage									
0	2 (100)	0 (0.0)	0.051	1 (50.0)	1 (50.0)	0.207	2 (100)	0 (0.0)	0.040
IA	10 (62.50)	6 (37.50)		15 (93.75)	1 (6.25)		16 (100)	0 (0.0)	
IIA	24 (77.42)	7 (22.58)		28 (90.32)	3 (9.68)		30 (96.77)	1 (3.23)	
IIIA	22 (68.75)	10 (31.25)		30 (93.75)	2 (6.25)		32 (100)	0 (0.0)	
IIB	4 (100)	0 (0.0)		3 (75.00)	1 (25.00)		3 (75.00)	1 (25.00)	
IIIB	3 (30.0)	7 (70.0)		10 (100)	0 (0.0)		10 (100)	0 (0.0)	
Her2-neo									
Negative	42 (71.19)	17 (28.81)	0.829	54 (91.53)	5 (8.47)	0.834	57 (96.61)	2 (3.39)	0.742
+	12 (60.00)	8 (40.00)		19 (95.00)	1 (5.00)		20 (100)	0 (0.0)	
++	4 (66.67)	2 (33.33)		5 (83.33)	1 (16.67)		6 (100)	0 (0.0)	
+++	7 (70.00)	3 (30.00)		9 (90.00)	1 (10.00)		10 (100)	0 (0.0)	

* Student *t*-test. +/3+, ++/3+, and +++/3+ represent standard positivity scores

**Table 3:** Age and Ki-67 association with inflammatory variables.

Variables	*IL-2* mRNA		*p* *	*IL-6* mRNA		*p* *	*TNF-α* mRNA		*p* *
	Absent	Present		Absent	Present		Absent	Present	
	Mean (CI 95%)			Mean (CI 95%)			Mean (CI 95%)		
Age	55.5 (52.2; 58.8)	59.7 (54.4; 65.07)	0.16	56.9 (54.0; 59.8)	55.6 (42.5; 69.2)	0.82	57.1 (54.3; 59.8)	46.5 (150.4; 243.4)	0.27
	Median (CI 95%)		*p* **	Median (CI 95%)		*p* **	Median (CI 95%)		*p* **
Ki-67 (%)	2.5 (0.0; 10.0)	2.0 (0.0; 15.0)	0.80	5.0 (0.0; 10.0)	0.0 (0.0; 30.28)	0.610	2.0 (0.0; 10.00)	17.5 (0.0; 35.00)	0.714

* Student *t* test. ** Kruskal–Wallis. CI 95%: confidence interval.

**Table 4:** Primers used in gene expression analysis.

Gene	Primers Sequence	Amplicon (bp)
*IL-2*	F-CCCAAGAAGGCCACAGAACT R-TTGCTGATTAAGTCCCTGGGT	125
*IL-10*	F-GCTGAGAACCAAGACCCAGA R-ATTCTTCACCTGCTCCACGG	141
*IL-6*	F-CCAGAGCTGTGCAGATGAGT R-AGCTGCGCAGAATGAGATGA	174
*TNF-α*	F-AGAGGGAAGAGTTCCCCAGG R-CCTCAGCTTGAGGGTTTGCT	123
*GAPDH*	F-GACCACAGTCCATGCCAT R-CAGCTCAGGGATGACCTT	148

## Data Availability

Data will be made available by correspondence author under reasonable request.

## Abbreviations

The following abbreviations are used in this manuscript:

BL1	Basal-like 1
BL2	Basal-like 2
DC	Dendritic Cells
ER	Estrogen Receptor
*GAPDH*	Glyceraldehyde-3-Phosphate Dehydrogenase
HER2	Human Epidermal Growth Factor Receptor 2
IDC	Invasive Ductal Carcinoma
*IL-2*	Interleukin-2
*IL-6*	Interleukin-6
*IL-7*	Interleukin-7
*IL-10*	Interleukin-10
*IL-12*	Interleukin-12
*IL-17*	Interleukin-17
ILC	Invasive Lobular Carcinoma
IM	Immunomodulatory
Ki-67	Proliferation Marker KI-67
LAR	Luminal Androgen Receptor
MSL	Mesenchymal Stem-like
mRNA	Messenger Ribonucleic Acid
*NF-κB*	Nuclear Factor kappa B
PCR	Polymerase Chain Reaction
PR	Progesterone Receptor
TCGF	T-cell Growth Factor
TNBC	Triple Negative Breast Cancer
*TNF-α*	Tumor Necrosis Factor Alpha
VEGF	Vascular Endothelial Growth Factor
